# Improving Multidimensional Wireless Sensor Network Lifetime Using Pearson Correlation and Fractal Clustering

**DOI:** 10.3390/s17061317

**Published:** 2017-06-07

**Authors:** Fernando R. Almeida, Angelo Brayner, Joel J. P. C. Rodrigues, Jose E. Bessa Maia

**Affiliations:** 1 PPGIA, University of Fortaleza (UNIFOR), Fortaleza 60811-905, Brazil; 2 Computer Engineering, Federal University of Ceará (UFC), Campus de Sobral, Sobral 62010-560, Brazil; 3 Department of Computer Science, Federal University of Ceará (UFC), Fortaleza 60440-900, Brazil; brayner@dc.ufc.br; 4 National Institute of Telecommunications (Inatel), Santa Rita do Sapucaí 37540-000, Brazil; joeljr@ieee.org; 5 Instituto de Telecomunicações, Universidade da Beira Interior, Covilhã 6201-001, Portugal; 6 ITMO University, St. Petersburg 197101, Russia; 7 University of Fortaleza (UNIFOR), Fortaleza 60811-905, Brazil; jmaia@unifor.br

**Keywords:** wireless sensor network, fractal clustering, multidimensional similarity measure, energy efficiency, multidimensional clustering, Pearson correlation

## Abstract

An efficient strategy for reducing message transmission in a wireless sensor network (WSN) is to group sensors by means of an abstraction denoted cluster. The key idea behind the cluster formation process is to identify a set of sensors whose sensed values present some data correlation. Nowadays, sensors are able to simultaneously sense multiple different physical phenomena, yielding in this way multidimensional data. This paper presents three methods for clustering sensors in WSNs whose sensors collect multidimensional data. The proposed approaches implement the concept of *multidimensional behavioral clustering*. To show the benefits introduced by the proposed methods, a prototype has been implemented and experiments have been carried out on real data. The results prove that the proposed methods decrease the amount of data flowing in the network and present low root-mean-square error (RMSE).

## 1. Introduction

Improvements in communication and sensor technologies have enabled the development of wireless sensor networks (WSNs) on a large scale. Sensors are tiny computers used to measure physical phenomena from the environment. Nonetheless, sensors suffer from restrictions in energy consumption, computational capacity, and memory area. WSNs are composed of several sensors spread in a given geographic region. Besides sensing data, sensors behave as nodes of the network. Thus, they are responsible for forwarding data in a multi-hop manner to a special node, denoted sink node or base station [1]. The sink node does not present restrictions regarding power consumption, computational power or memory area. In this work, we assume that the nodes of a WSN are grouped into clusters. Besides, the sink node is aware of the network topology with respect to the cluster configuration. Cluster configuration may be updated regularly to reflect possible changes in sensed data. Each sensor has the capacity to combine local and incoming data (from neighboring nodes). Hence, sensed data are transmitted from sensor to sensor until they reach a sink node.

Due to the characteristics of WSNs, they have been widely used for a plethora of applications, such as environmental monitoring, industrial sensing and diagnostics, infrastructure protection, and battlefield awareness, among others [2]. WSN applications can be categorized into three classes. Specifically, there are applications which: (1) submit queries to be processed by the network [3,4]; (2) collect all data sensed by sensor nodes (all the stream injected into the network); and (3) monitor sporadic events [5]. These types of applications are not mutually exclusive, i.e., in a given WSN, there may be applications submitting queries, applications consuming all data in the network and an application monitoring the occurrence of certain events.

Despite advances in wireless sensor networks technology, sensor energy consumption continues to be a critical issue [6]. This is because point-to-point messaging is notably the activity that most consumes energy in a WSN. Accordingly, efficient strategies to minimize energy consumption seek to decrease the number and the size of messages transmitted in the WSN. However, decreasing the amount of transmitted data may negatively impact the accuracy of the results provided by a sensor network. Hence, large accuracy rates usually come at the cost of high energy consumption.

It is well-known that sensor node clustering may decrease the amount of message transmission in a WSN, which in turn reduces energy consumption in the network. This is possible because, for each cluster *C*, a given sensor node, named cluster-head (CH), is responsible for collecting data sensed by the nodes that belong to *C* and forwarding these data to the sink. Moreover, a cluster-head may decide to forward only critical data to the sink. Many techniques have been proposed to create and to maintain clusters in WSNs [7,8,9,10,11]. Nevertheless, most of them use spatial correlation of sensed data as the main criterion for the clustering process.

The premise to cluster sensors in WSNs is to identify some correlation among data sensed by all sensors of the network or by groups of sensors. For instance, in WSNs data may be temporally and/or spatially correlated. In the former case, data obtained by a sensor in a given time window *T* may present a pattern. Thus, it is possible to compute future measurements (with a certain error threshold) by means of past measurements. For that, it is necessary to find an efficient regression model for inferring future data. In several scenarios, one can recognize that a group of sensors within a given spatial region *R* may be delivering similar values. Such a phenomenon, denoted spatial correlation, may occur due to the geographical proximity between the sensors belonging to such group. Hence, it is possible to infer values, which are being sensed in *R*, since sensor nodes are densely deployed in a WSN [1].

**Figure 1:**
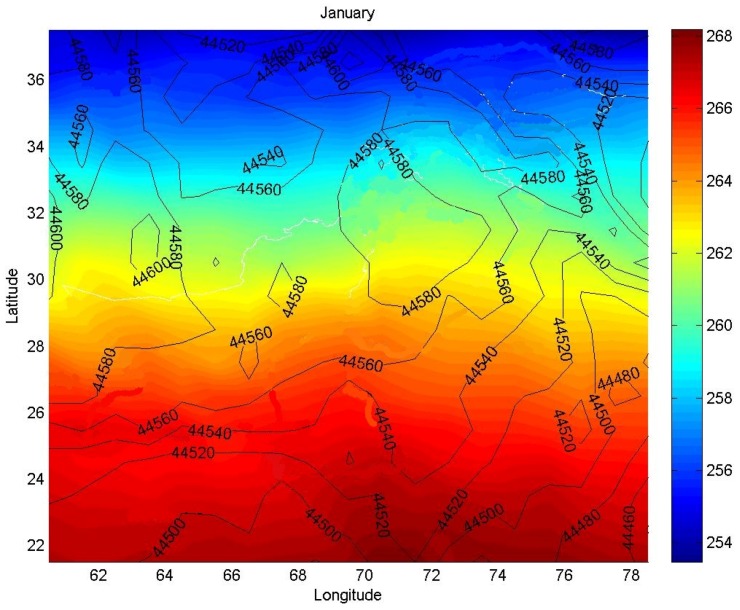
Temperature measures represented through different graduation of colors vs. luminosity contour lines.

Nonetheless, there are scenarios in which a group of sensors may present correlated measurements, even though they are spatially far apart from each other. Figure 1 illustrates an example of such scenarios. Figure 1 depicts luminosity contour lines vs. temperature measurements (represented by colors expressed in the bar on the right side of the figure), collected by sensors of a WSN for forest fire surveillance. The closed areas capture the fact that luminosity measurements collected by different sensors present some correlation, although those sensors are geographically distant from each other.

Looking more closely at Figure 1, one can observe areas 44,560 and 44,480. Sensors in those areas are collecting temperature and luminosity data which have some correlation. The proposed approach handles sensors in those different regions as they are in the same cluster. By doing that, only one cluster-head sends data from both regions to the sink node. Besides reducing the amount of messages injected into the network, the proposed approach provides data to the surveillance fire application more rapidly than conventional spatial correlation methods.

To capture data correlation among sensor nodes, which are not spatially close to each other, Rodrigues et al. [12,13] introduce the concept of *behavioral clustering*. By means of the notion of behavioral clustering it is possible to identify sensor nodes with similar data sensing patterns, even though they are far apart. Thus, sensors presenting behavioral correlation may be grouped into the same cluster.

Two approaches, namely *fractal clustering* and *similarity measure*, are proposed in [13] to cluster sensors which present behavioral correlation. However, the notion of *behavioral clustering* is applied only to one-dimensional data.

Multidimensional WSNs are deployed to sense data of different physical phenomena, where each physical phenomenon represents a dimension. For instance, in a WSN *w* with sensors being able to sense data on temperature, pressure, and humidity, one may say that *w* is a 3-D WSN. In a multidimensional WSN, sensors deal with data of different dimensions (physical phenomena). Thus, one can formulate the following hypothesis: clustering sensors using multidimensional data may yield more efficient clustering configurations with respect to the reduction of the amount of data inserted into the network.

In this paper, the concept of *behavioral clustering* is extended for clustering sensors in multidimensional WSNs. The main goal of *multidimensional behavioral clustering* is to automatically find correlated patterns of multidimensional data. By doing this, it is possible to dynamically initialize and maintain multidimensional sensor clusters.

The proposed multidimensional sensor clustering has three main phases: cluster initialization, data sensing and prediction, and cluster maintenance. In the first phase the initial cluster topology is constructed. The sensing and predicting phase is in charge to sense data and to send only outliers (or novelties) to the cluster-head node. The third phase is responsible for reconfiguring the cluster topology in order to capture new data patterns. All phases implement the concept of multidimensional behavioral clustering.

One may argue that the cluster initialization phase may demand high rates of power consumption. This is due to the fact that all sensors should send a certain amount of data of all sensed dimension. To reduce energy consumption during the initial phase, this paper introduces an approach, denoted *Pearson correlation with linear regression* (or PCLR, for short), for detecting multidimensional data correlation in WSNs by applying the Pearson correlation among single sensor node data. Moreover, the proposed approach utilizes linear regression coefficients to suppress sensed data from dependent dimensions. Thus, instead of transmitting the sensed data of all dimensions to the sink, only the data of one dimension, denoted the independent dimension, are injected into the network. If there are non-correlated dimensions, all the data from such dimensions should be transmitted as well.

The experiments show that PCLR can reduce the amount of data sent across the network up to 40%. Moreover, two novel techniques for maintaining and rebuilding the cluster topology are described. The first technique, denoted *fractal clustering in multidimensional WSN* (for short, FCM), implements the idea of *fractal dimension* [14] to dynamically build an optimal cluster configuration. The second one, named *similarity measure in multidimensional WSN* (SMM), exploits the notion of *multidimensional similarity measure* of sensed data (of different dimensions) to reconfigure cluster topology in WSNs. Both approaches aim at to dynamically adapt the cluster configuration according to the current data pattern (correlation).

Thus, the main contributions of this work may be summarized as follows:

- A novel approach for clustering sensor nodes based on the principle of multidimensional behavioral clustering is defined and analyzed;
- A novel technique (PCLR) is proposed. The key goal of PCLR is to diminish message transmission activity during the cluster initialization phase;
- Two different strategies (FCM and SMM) for verifying the efficiency of the current cluster topology are implemented. Both approaches are able to reconfigure the cluster topology on demand in order to achieve lower energy consumption rates in the network;
- An open-source simulator has been developed using SinalGo project [15]. The implemented simulator has been employed to run experiments on a real dataset (Intel Lab Data [16]).

The rest of the paper is organized as follows. Section 2 brings a discussion on related work. Section 3 presents and discusses the proposed approaches. Section 4 describes the experiments performed to evaluate the proposed approaches and analyses the obtained results. Finally, Section 5 concludes the paper.

## 2. Related Work

Over the last years, several approaches for reducing energy consumption in WSN have been proposed. Among them, sensor clustering has gained attention, since it can decrease power consumption and routing interval. In this section, sensor clustering approaches are described and analyzed. Furthermore, two approaches based on data prediction are described.

In spatiotemporal clustering and compressing schemes (SCCS) [9], the authors propose a sensor clustering mechanism based on spatial correlation of sensed data. The proposed mechanism yields the initial cluster configurations by triggering a flooding protocol. Thus, the sink node dispatches messages to all sensor nodes of the WSN, requesting data belonging to a given time window. Based on the received data, the sink node construct clusters by applying two metrics: dissimilarity measure and spatial distance among sensors. Thereafter, cluster-heads (CHs) are selected by taking into account the battery level. Sensor in a given cluster with the highest power level tends to be a CH. A round robin schedule for all clusters is implemented to specify which sensor node should be in operational mode in each time slot. Sensors in that mode should sense and send sensed data to the respective CH. The clustering process is the weak point of that work. This is due to high rates of communication activity in the WSN is required, which in turn implies in high power consumptions rates.

*Fractal clustering* (FC) is a method to cluster data sets proposed by Barbará and Chen in [14]. The first step of FC implements a k-nearest neighbor (k-NN) algorithm to create the initial cluster configuration. Afterward, it runs an incremental step algorithm, which computes fractal dimensions by means of the FD3 algorithm (an implementation of the box counting method) [17]. In this paper, we extend the FC approach by implementing a new version of the fractal clustering algorithm, executed during the cluster maintenance step (described in depth in Section 3.3.1). The extended algorithm avoids a high rate of message transmission among sensors belonging to a given cluster. The reason is that the algorithm, to compute fractal dimensions, requires only sensed data classified as novelties (outliers) to correctly compute the fractal dimensions. Besides, our approach applies an in-network data prediction model to avoid that all sensed data being transmitted to the sink node.

In [8], the authors propose a strategy to cluster sensors in WSNs. The idea is the following: given a set of N sensors, M nodes, with M<N, are chosen to send data. The M representative nodes are defined accordingly to the application of a distortion function (D(M)) on sensed data. The spatial distance between the nodes (representative) directly influences the computation of the distortion function by dint of a correlation coefficient. That work, named spatio-temporal correlation (STC), does not take into account the energy capacity of each node as a criterion for selecting a representative node in a cluster, although this is a critical factor in WSNs due to the restrictive characteristics regarding the energy consumption of nodes in a WSN. In the proposed approach, battery level is used only to define the cluster-head.

In [18], the authors propose a method to reduce energy consumption of sensors in a WSN, focusing on using sensors in a low *duty cycle*, which alternates between on and off (active and sleep) states, without disrupting the network operation. Two different approaches are evaluated: (1) random scheduling, which presents distributed sensors with independent random sleep (not centralized, nor coordinated); and (2) coordinated scheduling, which uses messaging among sensors to coordinate sleeping. The last approach is indicated to be better than the previous one. With a similar approach, in [19] the authors propose a node self-scheduling algorithm based on the eligibility rule for preserving coverage node in a WSN. In these two approaches, the main drawback is the assumption that the wireless sensor network has many sensors deployed in high density, and thus redundancy can be exploited.

Carvalho et al. proposed in [20] an approach, denoted multivariate spatio-temporal correlation (for short, MSTC), to improve data prediction accuracy in a WSN. However, that work does not reduce the energy consumption of the network. In fact, it even increases the network energy consumption in several situations. In our work, using multiple linear regression, we achieved the reduction of the overall energy consumption in the network, obtaining results with a smaller root-mean-square error (RMSE) for sensed data prediction.

Adaga-P* [2,21], an extension of the adaptive aggregation algorithm for sensor networks (Adaga) [22,23], constructs sensor-field models in the sink node. With a sensor-field model and a set of sensed data, Adaga-P* instantiates the model. Thus, Adaga-P* can diminish battery usage in sensor nodes, since they do not have to consume energy for building the model. Additionally, Adaga-P* implements a linear regression component to avoid having every sensed data item be transmitted to the sink node during the model instantiation. The prediction method can support the setup of several result-accuracy levels. Notwithstanding, it does not use the identified temporal correlation of sensed data to cluster sensors.

## 3. A Novel Sensor Clustering Mechanism

This paper proposes a sensor clustering mechanism for increasing multidimensional WSN lifetime. The proposed mechanism is more useful and suitable for WSNs having the following characteristics: it is multi-hop and centralized, with static nodes (non-mobile sensors) distributed in a random way in an outdoor environment and with ad-hoc queries, but mainly has multidimensional sensors, i.e., sensors that can read multiple physical phenomena simultaneously.

**Figure 2:**
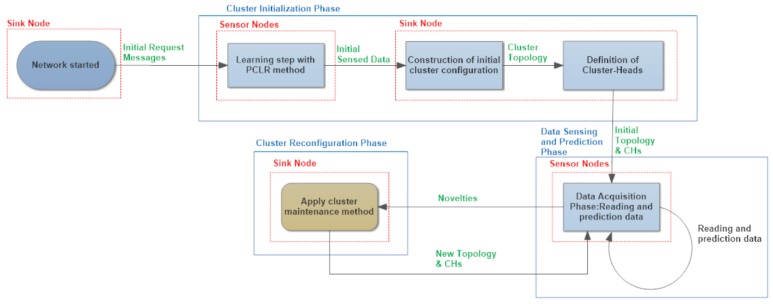
An abstract model of the proposed solution workflow. PCLR: Pearson Correlation of multidimensional data combined with linear regression coefficients; CH: cluster-head.

The proposed approach is composed of three phases: (1) cluster initialization; (2) data sensing and prediction; and (3) cluster reconfiguration. Figure 2 depicts a workflow, which describes the execution flow of those phases. The first phase, named cluster initialization, is executed in turn by three steps. The first step, denoted the learning step, starts when the sink node sends initial request messages (requiring multidimensional data) to all network sensors. Thus, sensor nodes collect multidimensional data during a given time interval. In order to minimize the number of sensed data sent by sensors to the sink node during the learning step, a novel technique, named PCLR, is executed at each sensor. By applying PCLR, sensor nodes send only sensed data of the independent variable and non-correlated variable, according to the Pearson correlation coefficient (see Section 3.1.1). The second step is executed in the sink node and has as an input parameter data sent by the sensors during the learning step. It is responsible for building an initial cluster configuration by applying the multidimensional similarity measure (MSM) method. Thereafter, in the third step, the sink node uses the cluster topology yield by the previous step to define the cluster-heads for the initial cluster configuration.

**Figure 3:**
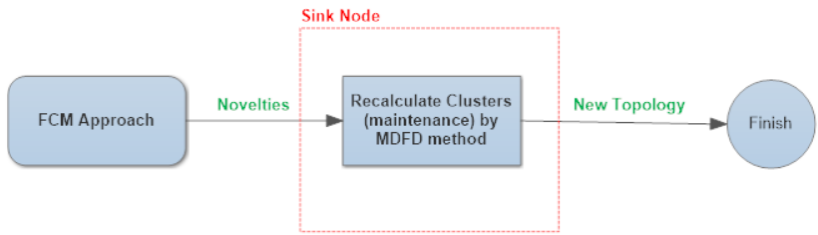
An abstract model of the proposed fractal clustering in multidimensional WSN (FCM) approach. MDFD: minimum difference of fractal dimension.

**Figure 4:**
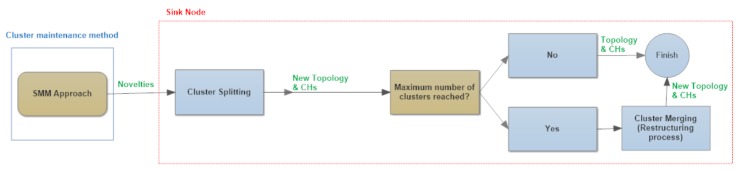
An abstract model of the proposed similarity measure in multidimensional WSN (SMM approach).

The second phase, the data acquisition phase, starts when sensors receive reading and predicting multidimensional data from the sink node. Periodically, the third phase is triggered. Two novel techniques are employed for cluster configuration maintenance, which are mutually exclusive in our solution (represented in Figure 3 and Figure 4). The choice is made by an application expert, depending on specific goals of the application. The first technique, denoted FCM, is responsible for maintaining the cluster topology by means of the *minimum difference of fractal dimension* (MDFD) factor, as one can see in Figure 3. The second one, named SMM, applies two different types of similarity, namely similarity of magnitude and similarity of trend. SMM is able to infer an efficient cluster topology with respect to the number of messages flowing in the network for a time slice.

The SMM approach implements two different maintenance actions: cluster split or cluster merge. SMM executes cluster split whenever novelties are collected by some nodes within a cluster. In this case, after the sink node proceeds with the cluster division, it checks if the number of clusters is above a certain maximum limit. In that situation, the sink node executes a global restructuring process (merge) with all network sensors (Figure 4). FCM and SMM are based on the notion of *multidimensional behavioral clustering*.

Next, the sensor clustering mechanism is described and analyzed.

### 3.1. Cluster Initialization

This phase can be further divided into three steps. Firstly, the sink requests data from each sensor belonging to the network. Each sensor reads the number of each data type requested and uses the Pearson correlation to identify the independent variable. Thus, each sensor sends data from the independent variable and the linear regression coefficients from dependent ones, plus the reading data from non-correlated variables. Thereafter, the sink constructs the initial cluster configuration and defines their corresponding CHs. Subsequently, the sink will determine the linear regression coefficients for each sensor and send the computed coefficients to all sensors.

#### 3.1.1. Learning Step: Pearson Correlation and Linear Regression

The main goals of this step are: (1) to construct the initial cluster configuration and (2) to compute the linear regression equation coefficients (see Section 3.2). For that, the sink node broadcasts a request to all sensor nodes, requiring them to sense and send back to the sink node data of all dimensions they should sense. The execution of this step requires two parameters: *initial slot time* (ist) and *data sensed types* (dst). The ist parameter represents a time window during which sensors should sense multidimensional data and send them back to the sink node. The size of ist determines the volume of sensed values used in the learning phase, since every sensor has a fixed sense range and knows the initial slot time parameter. In turn, dst identifies the dimensions of phenomena being sensed in the WSN. In other words, it represents the types of sensed data. Thus, the initial slot time and the dimensions of the WSN are parameters specified by an application expert.

During this step, all sensors work in a batch mode, which means each sensor should sense the defined amount of data and should send only one message to the sink. To make this feasible, the Pearson correlation coefficient (for short, PCC), also known as the Pearson product–moment correlation coefficient is computed for multidimensional data in a pairwise way. Hence, each sensor reads data of each required dimension and uses Pearson correlation to verify whether there exists a minimal correlation. The minimal correlation is a parameter defined by the application expert between the time series (set of data) belonging to the different data dimensions.

Therefore, through the PCC value *r*, a sensor node *S* can find out the independent variable. Note that the independent variable represents the data dimension with the maximum score, calculated as the sum of the r for all combinations of data dimensions which have the PCC value *r* greater than or equal to the minimal threshold (rPearsonMinimal), i.e. for that dimensions that have, at least, the minimum level of correlation to be considered correlated with the other.

For the sake of clarity, we next present important formulas, which formalize the proposed approach. They are helpful to understand specific terms used in this work.

For a multidimensional network, with *M* dimensions, let D[i] and D[j] be two dimensions, where 0<i,j<M+1, represent two distinct physical phenomena measured. In order to verify if there is a correlation between these two dimensions, the Pearson correlation coefficient is computed according to Equation (1), in a pairwise manner, for all *M* existing dimensions. The factor *N* represents the number of elements (readings in respective rounds) in each dimension (inside the specified time window ist) and it varies with *k*, in such a way that $D{\left[i\right]}_{k}$ and $D{\left[j\right]}_{k}$ are the corresponding current values in each dimension. Thus, data sensed by a given sensor *S* can represented by the following matrix:

$$S=\left[\begin{array}{ccccc} D{\left[1\right]}_{1} & D{\left[1\right]}_{2} & D{\left[1\right]}_{3} & \ldots & D{\left[1\right]}_{N}\\ D{\left[2\right]}_{1} & D{\left[2\right]}_{2} & D{\left[2\right]}_{3} & \ldots & D{\left[2\right]}_{N}\\ \vdots & \vdots & \vdots & \vdots & \vdots \\ D{\left[M\right]}_{1} & D{\left[M\right]}_{2} & D{\left[M\right]}_{3} & \ldots & D{\left[M\right]}_{N} \end{array}\right]$$

Matrix *S* has *M* lines, each of which represents the sensed dimensions (e.g., M=3 for three dimensions) and *N* columns, representing sensor readings (e.g., N=70 for 70 readings).

It is possible to generalize the sensed-data data of the *S* by $S=D{\left[m\right]}_{k}$, where m∈[1,M] and k∈[1,N]. Thus, the Pearson’s product–moment correlation coefficient (r) between any two dimensions *i* and *j* can be computed by the following formula:

(1) $$r_{D[i]D[j]}=\frac{\sum_{k=1}^{N}\left(D{\left[i\right]}_{k}-\bar{D[i]}\right)\left(D{\left[j\right]}_{k}-\bar{D[j]}\right)}{\sqrt{\sum_{k=1}^{N}{\left(D{\left[i\right]}_{k}-\bar{D[i]}\right)}^{2}}\sqrt{\sum_{k=1}^{N}{\left(D{\left[j\right]}_{k}-\bar{D[j]}\right)}^{2}}}$$

**Figure 5:**
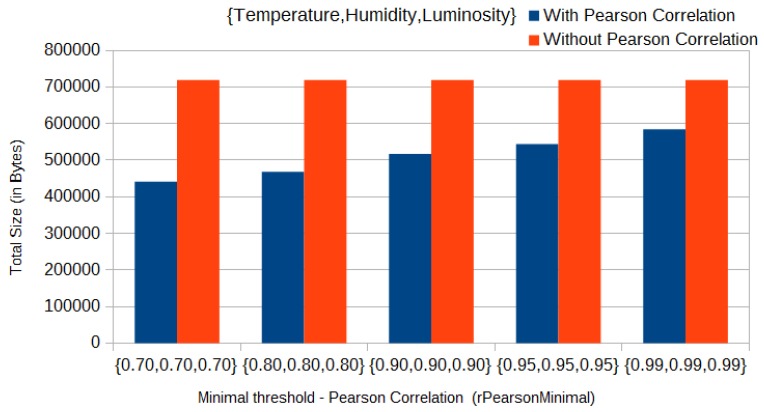
Comparison of data amount of messages sent––Pearson correlation with temperature, humidity and luminosity.

Furthermore, each sensor node *S* can compute the linear regression coefficients between each dependent variable and the independent one. Thus, a sensor *S* sends only sensed data of the independent variable and non-correlated variable. Sensed data of the dependent variables are not injected into the network, since only linear regression coefficients should be transmitted to the sink node. Such a strategy may reduce up to 40% the volume of data transmitted through the network, as one can see in Figure 5 (Section 4.2).

The use of linear regression is justified by the fact that this method of regression is sufficient to give the required precision of the obtained results and it is a simple method, of linear complexity. These are required features because sensors have restrictions on battery consumption and need to perform calculations light enough to get the longest possible lifetime. In addition, such a model has few parameters, consuming less memory and reducing the volume of data traffic on the network. The linear regression model has been extensively used with WSNs [1,2,3,7,12,13].

Let ind be the index of independent variable. Further, let *m* be a dependent variable, and $a_{m}$, $b_{m}$ are the linear coefficients calculated for the independent variable. $D{\left[ind\right]}_{k}$ represents the kth element of the independent variable (dimension ind). Thus, the sensed values, $\hat{D}{\left[m\right]}_{k}$, of a given dependent dimension (or variable) *m* can be computed by applying the linear regression equation expressed in Equation (2).

(2) $$\hat{D}{\left[m\right]}_{k}=a_{m}+b_{m}\cdot D{\left[ind\right]}_{k}$$

In Equation (2), parameter $a_{m}$ is the interceptor (value of $\hat{D}{\left[m\right]}_{k}$ for $D{\left[ind\right]}_{k}=0$) and $b_{m}$ is the stretch slope. The variables $b_{m}$ and $a_{m}$ are specified according to Equations (3) and (4), respectively.

(3) $$b_{m}=\frac{\sum \left(D{\left[ind\right]}_{k}-\bar{D[ind]}\right)\left(D{\left[m\right]}_{k}-\bar{D[m]}\right)}{\sum {\left(D{\left[ind\right]}_{k}-\bar{D[ind]}\right)}^{2}},$$

(4) $$a_{m}=\bar{D[m]}-b_{m}\cdot \bar{D[ind]}.$$

Consequently, the sink node is able to rebuild the complete multidimensional data set from all sensors, using the independent variable and the respective linear regression coefficients. That data set is used for computing initial clustering configuration to be handled by the sink in a centralized way. After the initial cluster formation and definition of cluster-heads, that data set is used as an input parameter to find the linear regression equation coefficients for each sensor node belonging to the WSN.

#### 3.1.2. Construction of Initial Cluster Configuration

For mounting the initial cluster topology, the MSM algorithm has been developed. The key idea behind that algorithm is to cluster sensors with similar multidimensional data reading patterns. The *multidimensional similarity measure* (based on [7]) between sensed data of two different sensors is defined by multidimensional magnitude similarity and multidimensional trend similarity, which are defined as follows:

**Definition 1** (Multidimensional magnitude similarity)**.** *Let S and $S^{\prime }$ be two sensors belonging to j-dimension WSN with following time series:*

S = {{$s_{1,1}$,$s_{1,2}$,…,$s_{1,N}$},{$s_{2,1}$,$s_{2,2}$,…,$s_{2,N}$},…,{$s_{j,1}$,$s_{j,2}$,…,$s_{j,N}$}} and
$S^{\prime }$ = {{$s_{1,1}^{\prime }$,$s_{1,2}^{\prime }$,…,$s_{1,N}^{\prime }$},{$s_{2,1}^{\prime }$,$s_{2,2}^{\prime }$,…,$s_{2,N}^{\prime }$},…,{$s_{j,1}^{\prime }$,$s_{j,2}^{\prime }$,…,$s_{j,N}^{\prime }$}}

*S and $S^{\prime }$ are multidimensional magnitude similar, if and only if ∀j>0, the following constraint is valid:*

(5) $$\bar{m}=\frac{\sum_{i=1}^{N}\left|s_{j,i}-s_{j,i}^{\prime }\right|}{N}\leq MM$$

In Equation (5), MM is a parameter provided by the application expert, while N is the amount of sensed data and j represents the number of data dimensions.

**Definition 2** (Multidimensional trend similarity)**.** *Let S and $S^{\prime }$ be two sensors belonging to j-dimension WSN with following time series:*

S = {{$s_{1,1}$,$s_{1,2}$,…,$s_{1,N}$},{$s_{2,1}$,$s_{2,2}$,…,$s_{2,N}$},…,{$s_{j,1}$,$s_{j,2}$,…,$s_{j,N}$}} and
$S^{\prime }$ = {{$s_{1,1}^{\prime }$,$s_{1,2}^{\prime }$,…,$s_{1,N}^{\prime }$},{$s_{2,1}^{\prime }$,$s_{2,2}^{\prime }$,…,$s_{2,N}^{\prime }$},…,{$s_{j,1}^{\prime }$,$s_{j,2}^{\prime }$,…,$s_{j,N}^{\prime }$}}

*S and $S^{\prime }$ are multidimensional trend similar, if and only if ∀j>0, the following constraint is valid:*

(6) $$\frac{P_{j}}{N}\geq MT$$

*In Equation (6), MT represents a parameter provided by the application expert, while N is the amount of sensed data and $P_{j}$ is the number of pairs $\left(s_{j,i},s_{j,i}^{\prime }\right)$ in the time series which satisfy the following condition:* *$\nabla s_{j,i}\times \nabla s_{j,i}^{\prime }\geq 0$, where $\nabla s_{j,i}=s_{j,i}-s_{j,i-1}$, $\nabla s_{j,i}^{\prime }=s_{j,i}^{\prime }-s_{j,i-1}^{\prime }$ and 1<i≤N, ∀j>0.*

#### 3.1.3. Definition of Cluster-Heads

As soon as the initial cluster topology is computed, the sink node starts the process defining the cluster-heads of each cluster. The premise for correctly executing is the sink must receive from each sensor the *battery level* and *spatial location*. The cluster-head selection process runs the following steps for each cluster:

1. The list of sensors belonging to the cluster is sorted in descending order by the battery level. If there are sensors with the same energy level, then the tiebreaker criterion is the shortest distance from candidate node to the sink;
2. The sensor node with largest energy level of battery and, if necessary, the smallest distance to the sink is chosen as the cluster-head.

### 3.2. Data Acquisition Phase

After the cluster-heads are defined, it is necessary to compute linear regression equation coefficients. Nonetheless, differently from the linear regression equation used to compute the Pearson correlation coefficient, the independent variable in this phase is time.

Thus, sensors and sink nodes have the same coefficients of linear regression equation for each type to estimate every sensed data of any data type. Accordingly, a sensor does not need to send all new reading data to the sink, because it is able to predict future data using the coefficients of linear regression equation. In this sense, sensors reduce energy consumption [2,21].

The sink node is in charge to calculate the initial coefficients (*a* and *b*) for each sensor and to forward them to the respective sensor node. As soon as a sensor node receives the corresponding linear regression coefficients, it starts to use the coefficients in the phase of reading and prediction loop. When at any time a sensor node reads a data value *v*, it checks if *v* is not inside an “allowed variation” *t*, i.e., if v∉[p−t,p+t],t≥0, let *p* be the estimated value by using the linear regression equation. Any data outside the allowable variation is treated as “novelty” by the model. “Allowed variation” is a variable set by the application expert (like 6% or 8%).

Whenever a sensor *s* identifies the existence of a given amount of novelties, sd (sensor delay) has been reached, and it sends the complete set of “new read data” as an alert to the CH of the cluster in which *s* is located. In turn, the CH logs the number of alerts. In such a way, when the number of received alerts is greater than a preset limit cd (cluster delay), the CH forwards a message to the sink that contains data of the newest readings, including battery level of the member sensors notifying the occurrence of novelties. When the sink gets this alert message, it begins the following phase, called *cluster maintenance*. The parameters sd and cd are set by the application expert.

### 3.3. Cluster Maintenance

Whenever the sink obtains an alert message (Section 3.2), it should perform two activities: (1) new regression coefficient computation; and (2) verification the *multidimensional behavioral clustering* holds true for the affected cluster.

The sink is in charge of computing new coefficients *a* and *b* for sensors belonging to the cluster that sent the novelties. For that, the sink node has to use the data, classified as novelties. The sink should store the new coefficients locally and send them to the corresponding sensors. Observe that *multidimensional behavioral clustering* among sensors must be ensured by the sink.

Correlation of sensed data may change in time. Such a feature demands the current cluster topology reconfiguration. Next, a detailed description of the proposed approaches (namely FCM and SMM) for cluster topology maintenance is presented.

#### 3.3.1. Cluster Maintenance with FCM Approach

The FCM approach is based on the notion of *fractal dimension* [14]. Its main goal is to identify the moment to reconfigure the current cluster topology. The idea is to monitor the amount of “novelties” received by the sink node. It is worthwhile mentioning that *fractal clustering* is an incremental clustering method. Hence, it is unable to create sensor clusters for the first time, requiring another algorithm to do so.

Algorithm 1 shows the way the FCM approach works. To describe Algorithm 1 it necessary to initially define critical variables. $R_{new}$ represents the collection of new sensed data forwarded to the sink node. Thus, $R_{new}$ contains “novelties” sensed by sensor node $S_{R}$. $C_{i}$ is the i−th cluster composed of a CH and other members (0 or more nodes). $D(C_{i})$ is the set of data collected in cluster $C_{i}$. In turn, $D^{\prime }\left(C_{i}\right)=D\left(C_{i}\right)\cup R_{new}$) (Line 5 in Algorithm 1). Finally, $FD(D\left(C_{i}\right))$ is the function which returns the magnitude of fractal dimension (FD) sensed data in cluster $C_{i}$.

In the initial clustering phase (Section 3.1.2), fractal dimension (FD) is computed ($FD(D\left(C_{i}\right))$) and saved for all initial clusters ($F_{d}Old\left[i\right],\forall i$) (Line 4 in Algorithm 1). The idea is to discover which cluster $C_{\hat{ı}}$ the insertion of new reading data $R_{new}$ will take to yield the smallest variation in its FD. Thus, from Line 3 (three) to Line 7 (seven) of Algorithm 1, old and new values of fractal dimension of each cluster $C_{i}$ are calculated and saved for comparison reasons.

**Algorithm 1:** Multidimensional fractal clustering algorithm—FCM approach.
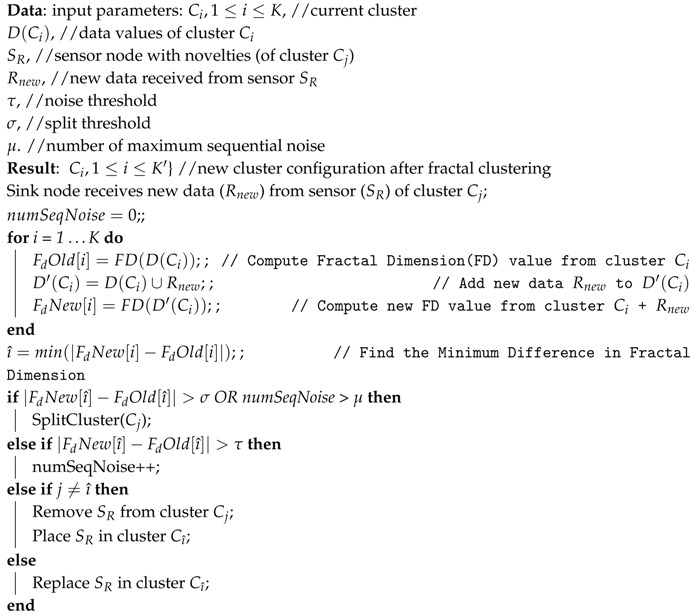


In Line 8 of Algorithm 1, *min*∇ returns the lowest values for $|F_{d}New\left[\hat{ı}\right]-F_{d}Old\left[\hat{ı}\right]|$, where $\hat{ı}=min(|F_{d}New\left[i\right]-F_{d}Old\left[i\right]\left|\right),0<i\leq K$). In the case *min*∇ is greater than threshold σ (e.g., σ=0.1) or if the consecutive noise counter (numSeqNoise parameter) is larger than a maximum amount of consecutive noise (μ) (defined by the application expert), then a cluster split operation is triggered (Lines 9–10). On the other hand, in the case *min*∇ is higher than a given threshold (τ), new sensed data belonging to $R_{new}$ is discarded, since they are classified as noise. In this case, the successive noise counter (numSeqNoise) is incremented for the current sensor node (Lines 11–12).

If i=j, the sink node computes new coefficients (*a* and *b*) for sensor nodes in this cluster. Alternatively, the sink should eliminate this node ($S_{R}$ with new sensed data $R_{new}$) from its last cluster $C_{j}$ and append it to the new cluster $\left(C_{i}\right)$, as we can see from Lines 13–18 from Algorithm 1. It is important to highlight that the proposed multidimensional fractal clustering approach focuses on fractal dimension of the data sensed of sensors, but not on sensor node physical location, as in [24].

#### 3.3.2. Cluster Maintenance with the SMM Approach

Cluster maintenance in the SMM approach uses the same method for building the initial cluster configuration, described in Section 3.1.2. However, for the cluster maintenance phase, SMM is composed of the following activities: cluster division and cluster merging.

1. Cluster division (or splitting)––The cluster division process will be controlled by the sink node and this process will be started at any time it receives novelties from some CH and it observes the set of sensor nodes from that cluster is no longer complying with the minimal similarity requirements needed to maintain that sensors grouped (as one can see at Section 3.1.2). In that situation, the sink should fire the cluster splitting process of the given cluster, choosing the CHs for each one of new clusters, according to that described in Section 3.1.3. Additionally, the sink must keep control of the maximum number of clusters, in such a way that it is capable of firing the “restructuring process” (or merging), if there are many divisions.
2. Cluster merging (restructuring process)—When a high amount of cluster splitting occurs and the minimum rate occupation of the clusters (average of nodes per cluster) on the network is disregarded, the sink should start a restructuring (merge) process involving the entire network, such that all CHs should require that sensors read data from all nodes to be sent to and reassessed by the sink, and thus, the clusters can be reconfigured into new ones that better represent the current state of the network. In this manner, one avoids that in time, an increasing number of divisions (splits) of network clusters may generate small clusters, expanding the energy expenditure of the network’s sensors.

In Line 1 of Algorithm 2, a boolean function (called *TestSimilarityMeasure*) is declared, with the goal of testing if two given simultaneous multidimensional data readings of two distinct sensor nodes have similar measures or not. In this way, a comparison will be made as to whether magnitude (Lines from 5 to 7) and trend (Lines from 8 to 10) are both similar (Line 11).

Lines 13–15 describe the requirements of the sink node to fire the SMM approach. Thus, in Line 16, a flag (*ExistingClusterFound*) is set to *false*, as default. Next, for each cluster $C_{k}$ for which the respective CH sends a notification (“novelty”) message to the sink, the sink will receive new multidimensional reading data and it will use the *TestSimilarityMeasure* function to verify if the current sensor data is similar to all sensors from some clusters (Lines 17–24).

If the multidimensional reading data of the current sensor node is not similar to any current cluster (*ExistingClusterFound = false*), then a new cluster is created (Cn) and the respective sensor node is added to it (Lines 25–28).

**Algorithm 2:** Similarity measure multidimensional algorithm—SMM approach.
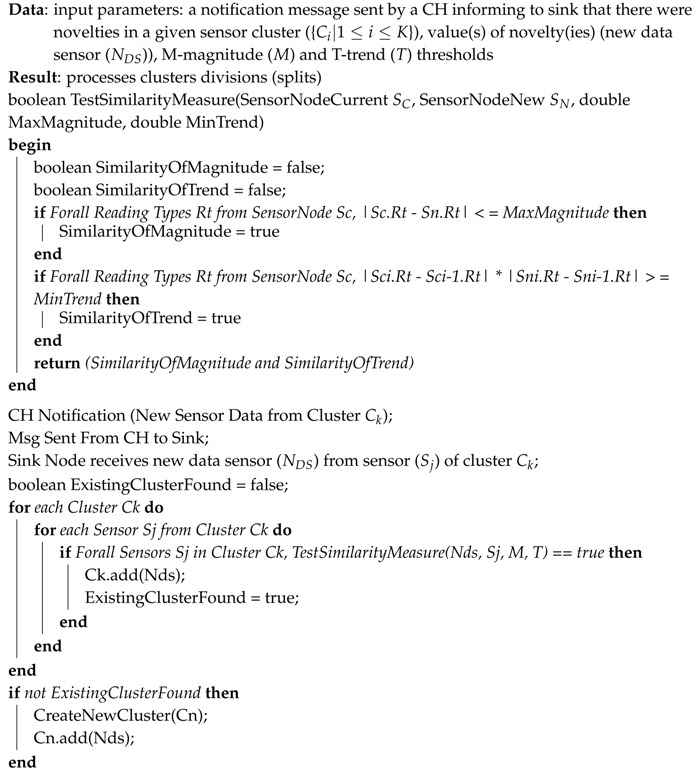


### 3.4. Discussion

One of the main issues in wireless sensor networks is sensor node synchronization. Nevertheless, the proposed methods do not require synchronization among the sensors, since it is enough to have the number of sensors in the network and the amount of sensed data specified by a given computation. Therefore, the sink node knows when it has received all initial data from sensors. Moreover, there is a read counter (round Id) for each sensor.

Another important question is with respect to the described threshold values. As a matter of fact, those values have been obtained empirically with a fine-tuning of such variables. However, it is important to assert that those parameters do not need to change over time.

## 4. Experiments and Results Analysis

For showing the potential of these approaches, a series of empirical tests was executed over real data and results achieved are presented and discussed in this section. The main question is as to which approach is more appropriate to reduce energy consumption and which is most suitable to maintain improved accuracy, while multidimensional data reading sensors were used.

### 4.1. Implementation and Simulation Setup

A simulation prototype has been implemented in Java, inside Sinalgo [15], a well-known framework used for testing and validating WSN algorithms. Experiments have been performed by a machine with a 2.8 GHz Intel Core i7 processor, 8GB DDR3 RAM and running Mac OS X 10.10.3.

One of the main reasons for choosing Sinalgo is related to its scalable nature. Moreover, it has the necessary support for the planned experiments in which the use of thousands of sensor nodes is required. For evaluating the proposed approaches, a real dataset has been used (provided by the Intel Berkeley Research Laboratory [16]).

In this section, we present the results obtained in our experiments. First, we present and discuss the results of running the Pearson correlation in combination with the linear regression (see SubSection 4.2) during the initial sensor clustering phase. Thereafter, we compare two methods to yield the initial cluster configuration: k-nearest neighbors (k-NN) and multidimensional similarity measure (MSM). Finally, we describe the results of using FCM and SMM approaches (SubSection 4.4).

### 4.2. Pearson Correlation and Linear Regression

The Pearson correlation coefficient (r) shows the magnitude of linear correlation between any two variables. This way, one can measure the strength of a correlation between variables across this coefficient, using the following interpretation: (1) r=0.9 indicates a very strong correlation; (2) *r* between 0.7 and 0.9 positive or negative indicates a strong correlation; and (3) *r* below 0.7 indicates a moderate, weak or irrelevant correlation depending on the value of r [25]. Thus, in our simulations, we consider the existence of correlation when the Pearson correlation coefficient (r) is greater than or equal to 0.7.

The Pearson correlation algorithm implementation considers that the independent variable will be the one that, having calculated the Pearson coefficients for all variables pair to pair, has the largest sum of coefficients greater than the minimum threshold (in our tests, the minimum threshold—called *rPearsonMinimal*—was set for the following values: 0.7, 0.8, 0.9, 0.95 and 0.99).

For each sensor in the network with the choice of the independent variable ’x’, we use ’x’ to calculate the linear regression coefficients ’a’ and ’b’ between ’x’ and each of the dependent variables of that sensor. Those variables that happened to have the Pearson correlation coefficient, relative to the independent variable, lower than the minimum threshold (0.7) are considered non-correlated variables. Thus, our solution can suppress the transmission of complete set of values (time series) from dependent variables, replacing the data set only for the respective linear regression coefficients (’a’ and ’b’), that will be used later to reconstruct the reading data from the dependent variables in sink node [20]. This method has proved to be effective in filling the gaps of readings not made by sensor failures.

**Figure 6:**
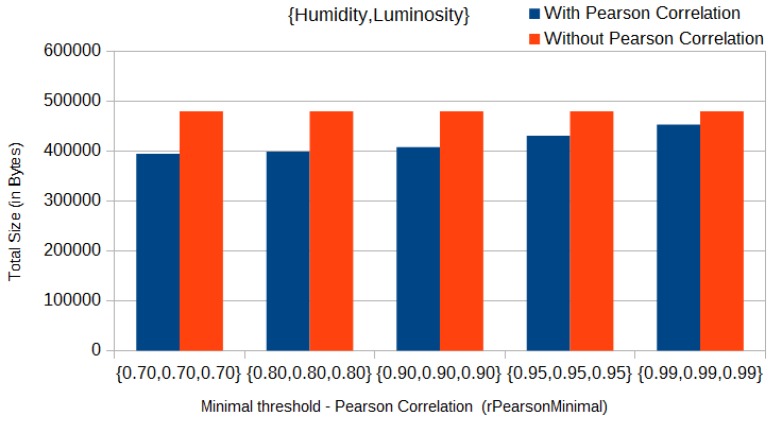
Comparison of data amount of messages sent—Pearson correlation with humidity and luminosity.

In our experiments, we used multidimensional data of the three physical phenomena. In that case, we considered an initial set of data of size equal to 70 readings from each of the sensed data types, to be sent by each sensor to the sink. Thus, with the Pearson correlation approach in use, the total size of messages sent by each sensor in the initial clustering phase decreased from 719 kB to 441 kB, that is, approximately 40% less network traffic in the initial phase, when using the three physical phenomena and with a threshold (*rPearsonMinimal*) of 0.7 for all parameters (Figure 5). On the other hand, in a worst-case scenario, when using only humidity and luminosity and with a threshold (*rPearsonMinimal*) of 0.99 for both parameters, the total size of messages sent in the initial clustering phase decreased from 479 kB to 452 kB (approximately 5%), as we can see in Figure 6.

### 4.3. Initial Clustering: k-NN vs. MSM

For multidimensional clusters initialization, the sink requests a certain amount of multidimensional sensed data from each sensor, which are sent to the sink. After receiving the requested data, the sink needs to classify each sensor into clusters (comparing multidimensional sensed data) and define the clustering configuration using *multidimensional behavioral clustering* of sensed data.

*Fractal clustering* is an incremental clustering technique. Hence, it is unable to initialize sensor clusters, requiring another algorithm able to do so.

In this work, we compared two different methods to define the initial clustering configuration: the general well-known k-nearest neighbors (k-NN) algorithm and the specific multidimensional similarity measure (MSM) algorithm. Parameter values for both algorithms were empirically defined. For k-NN, the *k* value was, empirically, fixed at 16, and the parameter values for MSM were MM=1.5 and MT=0.05.

Our tests showed that the specialized MSM is better than the general k-nearest neighbors (k-NN), because keeping other parameters values, and using the FCM approach to cluster maintenance, we obtained the following results (see Table 1) for the number of messages exchanged by the sensors, when we only changed the initial clustering method between k-NN and MSM.

Besides that, Table 2 shows the RMSE for the FCM approach by using MSM compared with k-NN (NN with k = 2) as initial cluster algorithms for gathering *temperature* data. Observe that using k–NN as initial cluster method, the RMSE is greater than same approach using MSM for all observed measurements.

### 4.4. Maintenance of Clusters: FCM vs. SMM

With the goal of evaluating the proposed cluster maintenance approaches, two metrics have been employed: root-mean-square error (RMSE) to assess the precision of approaches, and amount of messages injected into the network. *Temperature*, *humidity* and *luminosity* were the dimensions used in the experiments, with the following combinations: (1) only *humidity*; (2) only *luminosity*; (3) *temperature* and *humidity*; and, finally; (4) *temperature*, *humidity* and *luminosity*.

Table 3 shows the generic simulation parameters and their corresponding values. The specific simulation parameters for SMM methods, with their values, are presented in Table 4. In Table 3, the number of initial readings parameter represents the amount of readings that all sensors sent to sink node during the learning phase. Multidimensional magnitude and multidimensional trend were defined in Section 3.1.2. Allowed variation is the acceptable threshold error. Sensor delay parameter means the number of prediction errors each sensor may accept before forwarding an alert to its CH. In analogous way, cluster delay parameter means the number of alerts all CHs may tolerate before sending a notification to the sink node.

Firstly, we present the results for *humidity* data gathering to show the efficiency of the proposed approach with one dimension. Table 5 shows root-mean-square error (RMSE) values for FCM and SMM approaches at given numbers of cycles—namely, cycles 250, 500, 750, and 1000. RMSE is calculated by reference to the naive approach filtered values. On the other hand, Table 6 shows values for the number of messages sent per cycle (with the average (AVG), standard deviation (STD), maximum (MAX), and minimum (MIN)) from the two approaches evaluated during the experiments.

Observing Table 5 and Table 6, one can observe that FCM presents a lower RMSE than SMM. Nevertheless, the average number of transmitted messages in SMM is smaller than the FCM approach (see Table 6). In this case, with respect to the number of transmitted messages, the SMM approach provided better results, with a lower number of messages.

It is worthwhile to note that in Table 6, the values of column MIN for FCM and SMM have the same value (zero). This is due to the fact that the calculated value in several cycles (see Section 3) is very similar to the sensed value. In this situation, the sensor node does not demand to forward the sensed data.

**Figure 7:**
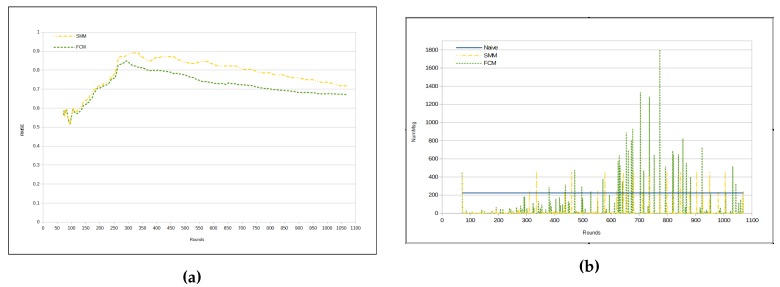
Results—humidity. (**a**) RMSE; (**b**) Number of messages for each cycle.

Figure 7a depicts evolution of RMSE per round. Observe that FCM and SMM RMSEs begin quite similarly. However, from a certain point in time on (approximately from round 110), they begin to present a different behavior, with an increase in the difference in round 260. From that point on, the FCM approach has a lower RMSE than the SMM approach (Table 5).There is a peak for the both FCM and SMM lines, between rounds 300 and 350. The reason for that is the fact that the first cluster formation represents the initialization step for both approaches. Hence, both FCM and SMM accuracies are jeopardized at the beginning. Nevertheless, FCM and SMM approaches are able to automatically adjust the accuracy by reducing the RMSE through the use of *fractal clustering* or *similarity measure*, correspondingly. It is important to note that in the naive approach the RMSE is virtually 0 (zero). This is because all sensors send all sensed data to the sink.

Figure 7b presents the number of transmitted messages per round. In the naive approach, the number of messages is constant, since in each round all sensors send all sensed data to the sink. It is worth noting that there are some peaks in FCM and SMM curves, which represent periods in time, when clusters are being restructured (splitting or merging). Recall that these approaches (FCM and SMM) dynamically update the cluster configuration. Such a characteristic of the proposed approaches is responsible for the high standard deviation in Table 6.

**Figure 8:**
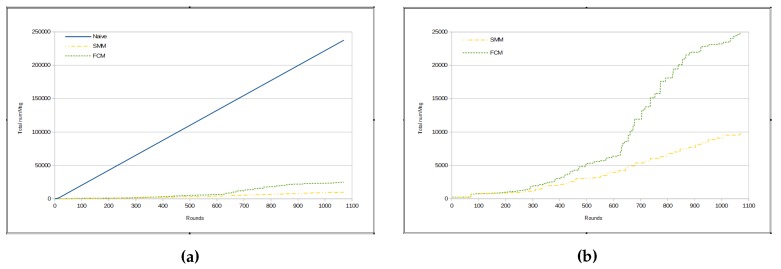
Total number of messages—humidity. (**a**) naive × SMM × FCM; (**b**) SMM × FCM.

It can be inferred that the amount of messages sent per round in FCM approach is, mostly, bigger than in SMM. In fact, the total number of messages in the FCM approach has been shown to be much greater than the number of messages in SMM for *humidity* measurements, as we can see in Figure 8a,b. Figure 8a presents a comparison among the total amount of messages from three approaches, namely naive, FCM and SMM. The total number of messages sent by sensors in naive approach is much greater than for the other two approaches, because in the previous method all sensors send all sensed data through messages immediately to the sink, whereas in other approaches there are some kinds of data suppression.

Next, we show the results obtained for the *luminosity* dimension, to compare a highly volatile (i.e., unpredictable) data type (such as *luminosity*), with a more predictable one (such as *humidity*).

As we can see in Table 7, the RMSE values for *luminosity* reveal that this physical phenomena has a very large variation, making it highly unpredictable. However, unlike the case of *humidity*, in this situation the RMSE values for SMM approach were lower than for the FCM approach.

Despite the high number of messages compared to reading the *humidity*, SMM approach again had the best results compared to FCM, in relation to the average number of sent messages per cycle for *luminosity* data type (Table 8).

**Figure 9:**
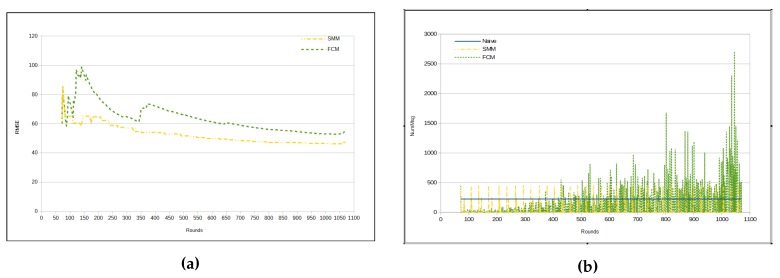
Results—luminosity. (**a**) RMSE; (**b**) Number of messages for each cycle.

Figure 9a shows that when the RMSE of both approaches stabilizes, SMM has lower error than FCM approach. The number of messages per cycle starts smaller for FCM in comparison with SMM approach, whereas from about round 400 the FCM approach has significant peaks, reversing the situation presented until that point (Figure 9b). This is due to the fact that the FCM approach deals with one sensor cluster reorganization per time, whereas the SMM approach makes a global merging process from time to time. As luminosity is highly unpredictable, a side effect is the shown high message exchange.

**Figure 10:**
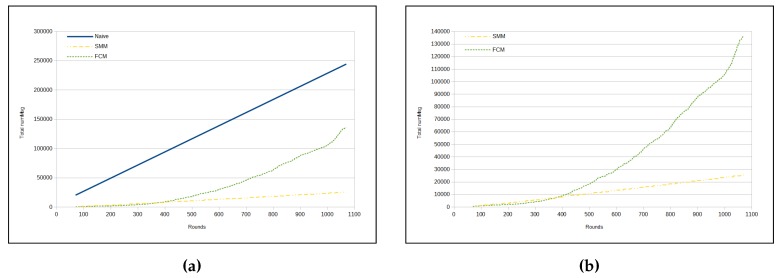
Total number of messages—luminosity. (**a**) naive × SMM × FCM; (**b**) SMM × FCM.

The total number of sent messages in both approaches is smaller than in the naive, mainly in the SMM approach. However, even for the FCM approach, the number of messages is approximately half that of the naive (see Figure 10a). On the other hand, Figure 10b shows that the SMM sends a smaller amount of messages than FCM from round 400 onwards.

Now, we compare the proposed approaches using two dimensions simultaneously, namely *temperature* and *humidity*.

**Figure 11:**
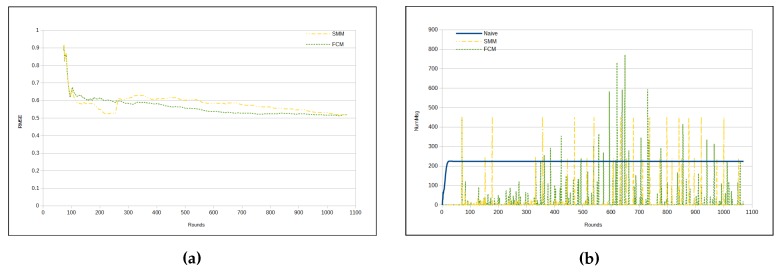
Results—temperature and humidity. (**a**) RMSE; (**b**) Number of messages for each cycle.

**Figure 12:**
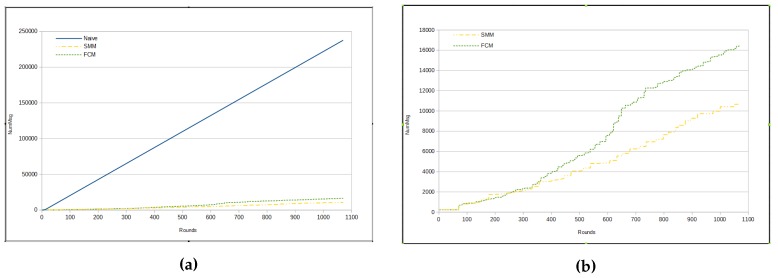
Total number of messages—temperature and humidity. (**a**) naive × SMM × FCM; (**b**) SMM × FCM.

Table 9 presents the results for RMSE, while using *temperature* and *humidity* as sensed data. As one can see, RMSE in this case is very similar to the cases with only one sensed data. As a matter of fact, RMSE is better in this case than it is with *humidity* as unique sensed data. Comparing the two proposed approaches presented in Table 9 and Table 10, the relation between the approaches is maintained, in which the FCM approach presents a smaller RMSE than the SMM approach (Figure 11a), and, in turn, the SMM injects a smaller number of messages in the WSN than the FCM approach (as in Figure 11b, Figure 12a,b).

Finally, we evaluate the proposed approaches using three dimensions at the same time: *temperature*, *humidity* and *luminosity*. The results show the ability of the proposed approaches to handle with multiple dimensions simultaneously.

In spite of the participation of *luminosity* as data type, one can observe in Table 11 that *temperature* and *humidity* together with *luminosity* yield a RMSE reduction in comparison with *luminosity* results (see Table 7).

Comparing Table 12 with Table 8, we can see that the average number of messages per round in FCM approach for three data types (including *luminosity*) is slightly larger than that for *luminosity* only. However, the same parameter in SMM approach is virtually equal for the two cases. It is important to note that SMM approach presents an average number of messages much smaller than that of FCM approach in both situations.

**Figure 13:**
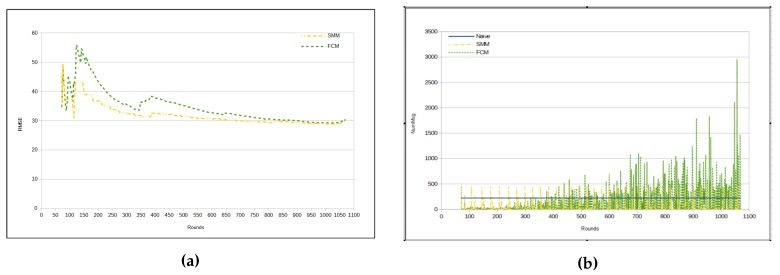
Results—temperature, humidity and luminosity. (**a**) RMSE; (**b**) number of messages for each cycle.

Figure 13a presents a RMSE curve from the two approaches (namely SMM and FCM) applied to three data types with a behavior very similar to same approaches applied only to *luminosity*, as one can see in Figure 9a. However, it is important to note two major differences: (1) the magnitude of RMSE values, which in this situation is approximately half of the values of the previous case; and (2) the relative difference between the RMSE numbers for two approaches from round 800 onwards, which drops the difference virtually to zero, making the two approaches present very similar RMSEs.

**Figure 14:**
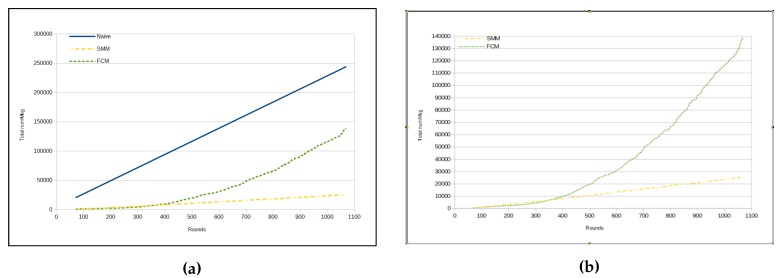
Total number of messages—temperature, humidity and luminosity. (**a**) naive × SMM × FCM; (**b**) SMM × FCM.

With regard to the total number of messages sent, we can observe that there is a great similarity between the results made only with the *luminosity* data type (Figure 9b and Figure 10a,b) and those made with the data types *temperature*, *humidity* and *luminosity* (Figure 13b, Figure 14a,b).

Up to a point, for each graph showing the total number of messages for the two compared approaches (as in Figure 8a, Figure 10a, Figure 12a and Figure 14a), we made another graph which did not include the naive curve for the total number of messages (Figure 8b, Figure 10b, Figure 12b and Figure 14b), because it is too high due to the other two approaches.

## 5. Conclusions

This paper presented a novel mechanism for clustering sensors in multidimensional WSNs, which implements the notion of *multidimensional behavioral clustering*. The proposed mechanism incorporates a technique, which combines Pearson correlation and linear regression, for reducing communication activity during the initial phase. Additionally, two novel techniques are employed to group sensors in a multidimensional WSN. Both techniques are based on the principle of *multidimensional behavioral clustering*.

*The Pearson correlation* and the use of linear regression coefficients were demonstrated to be efficient solutions for reducing the message transmission in the learning phase. This is because only data of the independent dimension are sent to the sink node. Furthermore, two techniques for maintaining the cluster topology were presented. The first technique, named FCM, uses the principle of *fractal dimension* to discover on-the-fly a good configuration for clusters in a given time window. In turn, SMM uses *multidimensional similarity measure* for clusters maintenance in WSNs.

Section 4 presented results showing that the proposed methods can significantly reduce energy consumption in multidimensional WSNs. This because the proposed approaches lower the number of messages sent through the network. Besides, they indicate that the proposed approaches assure low RMS error rates.

Therefore, it can be stated that the FCM method should be applied in scenarios in which multidimensional WSN application demand to obtain a given energy saving and low RMSE is crucial for successful deployment of the WSN. In all other situations, the SMM method represents a good trade-off between energy saving and precision (low RMSE) for data-gathering in a multidimensional WSN application. In conclusion, the appropriateness and the efficiency of our multidimensional methods should be highlighted for a bulk of applications that use multidimensional data originating from multidimensional WSNs.

## Figures and Tables

**Table 1:** k-nearest neighbors (k-NN) vs. multidimensional similarity measure (MSM): NumMessages.

#Msg × Cycle	250	500	750	1000
k-NN	6539	34343	81955	150585
MSM	5429	28446	78283	137573

**Table 2:** FCM root-mean-square error (RMSE) by initial clustering method—temperature.

RMSE × Cycle	250	500	750	1000
FCM using MSM	0.464	0.401	0.396	0.39
FCM using k-NN (k = 2)	0.826	0.764	0.72	0.693

**Table 3:** Generic simulation parameters.

Parameter	Value(s)
Sensed data types	Temp./Hum./Lum.
Number of initial readings	70
Multidimensional magnitude *(MM)*	1.5
Multidimensional trend *(MT)*	0.05
Allowed variation *(t)*	5%
Sensor delay *(sd)*	1
Cluster delay *(cd)*	5

**Table 4:** SMM simulation parameters.

Parameter	Value
Multidimensional magnitude	1.5
Multidimensional trend	0.05
Minimum occupancy rate per cluster	1.35

**Table 5:** RMSE—humidity.

RMSE × Cycle	250	500	750	1000
FCM	0.823	0.739	0.698	0.672
SMM	0.890	0.846	0.775	0.718

**Table 6:** Number of messages—humidity.

Num Msg	AVG	STD	MAX	MIN
FCM	24.569	125.308	1805	0
SMM	9.589	55.2283	450	0

**Table 7:** RMSE—luminosity.

RMSE × Cycle	250	500	750	1000
FCM	63.311	62.588	55.861	54.413
SMM	57.06	50.526	47.113	47.172

**Table 8:** Number of messages per round—luminosity.

Num Msg	AVG	STD	MAX	MIN
FCM	135.990	258.6486	2710	0
SMM	25.245	93.8936	450	0

**Table 9:** RMSE—temperature and humidity.

RMSE × Cycle	250	500	750	1000
FCM	0.58	0.542	0.525	0.519
SMM	0.627	0.588	0.557	0.518

**Table 10:** Number of messages per round—temperature and humidity.

Num Msg	AVG	STD	MAX	MIN
FCM	16.176	66.1338	772	0
SMM	10.424	57.3927	450	0

**Table 11:** RMSE—temperature, humidity and luminosity.

RMSE × Cycle	250	500	750	1000
FCM	34.527	33.32	30.488	30.295
SMM	32.331	30.838	29.68	29.58

**Table 12:** Number of messages per round—temperature, humidity and luminosity.

Num Msg	AVG	STD	MAX	MIN
FCM	138.063	265.5481	2954	0
SMM	25.241	93.8797	450	0

## Supplementary Materials

The full project and source code of the developed simulator using SinalGo project is available online at https://github.com/fernandoraj/Cluster_WSN.
